# Short-Term Effects of the Particulate Pollutants Contained in Saharan Dust on the Visits of Children to the Emergency Department due to Asthmatic Conditions in Guadeloupe (French Archipelago of the Caribbean)

**DOI:** 10.1371/journal.pone.0091136

**Published:** 2014-03-06

**Authors:** Gilbert Cadelis, Rachel Tourres, Jack Molinie

**Affiliations:** 1 Department of Pulmonary Medicine, Universitary Hospital of Pointe-a-Pitre, Pointe-a-Pitre, Guadeloupe, French West Indies; 2 Laboratory of Research in Geoscience and Energy, University of Antilles and Guyane, Pointe-a-Pitre, Guadeloupe, French West Indies; University of Cincinnati, United States of America

## Abstract

**Background:**

The prevalence of asthma in children is a significant phenomenon in the Caribbean. Among the etiologic factors aggravating asthma in children, environmental pollution is one of the main causes. In Guadeloupe, pollution is primarily transported by Saharan dust including inhalable particles.

**Methods:**

This study assesses, over one year (2011), the short-term effects of pollutants referred to as PM_10_ (PM_10_: particulate matter <10 µm) and PM_2.5–10_ (PM_2.5–10_: particulate matter >2.5 µm and <10 µm) contained in Saharan dust, on the visits of children aged between 5 and 15 years for asthma in the health emergency department of the main medical facility of the archipelago of Guadeloupe. A time-stratified case-crossover model was applied and the data were analysed by a conditional logistic regression for all of the children but also for sub-groups corresponding to different age classes and genders.

**Results:**

The visits for asthma concerned 836 children including 514 boys and 322 girls. The Saharan dust has affected 15% of the days of the study (337 days) and involved an increase in the average daily concentrations of PM_10_ (49.7 µg/m^3^ vs. 19.2 µg/m^3^) and PM _2.5–10_ (36.2 µg/m^3^ vs. 10.3 µg/m^3^) compared to days without dust. The excess risk percentages (IR%) for visits related to asthma in children aged between 5 and 15 years on days with dust compared to days without dust were, for PM_10, (_(IR %: 9.1% (CI95%, 7.1%–11.1%) versus 1.1%(CI95%, −5.9%–4.6%)) and for PM_2.5–10_ (IR%: 4.5%(CI95%, 2.5%–6.5%) versus 1.6% (CI95%, −1.1%–3.4%). There was no statistical difference in the IR% for periods with Saharan dust among different age group of children and between boys and girls for PM_10_ and PM_2.5–10_.

**Conclusion:**

The PM_10_ and PM_2.5–10_ pollutants contained in the Saharan dust increased the risk of visiting the health emergency department for children with asthma in Guadeloupe during the study period.

## Introduction

The prevalence of asthma, especially in children, increased in the world over the last decade [1]. In the Caribbean, this disease represents a preoccupying public health problem [2]. In Guadeloupe (16° North latitude and 61° West longitude), the prevalence of asthma in children is higher than in France (14% versus 9%) [3]. Among the many etiologic factors causing asthma in children, pollution, especially particulate pollution, plays an important part [4].

The archipelago of Guadeloupe is periodically exposed to Saharan dust generating peak exposures to fine particles, which can last several days. This particulate pollution contributes to exceeding the particle thresholds set in relation to health protection. The desert sand dust is generated above the Sahara desert. The production of dust is at its highest level between April and June. It is assessed at around 500 to 1000 tonnes per year [5]. The dust particles, captured by the winds at the ground surface, are driven to tropospheric altitudes. These particles are transported as suspended matter, at an altitude between 1500 and 6000 m of the African desert, towards the West, over the Atlantic Ocean, and they reach the United States due to the influence of the maritime trade winds. They therefore pass via the Caribbean, between April and October, and settle through wet or dry processes [6]. The granulometric measurements performed on the particles, at more than 100 km from the dust source, showed that the median diameter rapidly decreases below 10 µm [7]. During a dust episode, the concentration in particles can reach 2000 µg/m^3^ as a maximum hourly average [7]. The majority of the dust particles measure less than 10 µ/m of aerodynamic diameter and are thus inhalable, as the smallest particles can easily penetrate inside the respiratory tracts and therefore reach the bronchi and the small airways. The Saharan particles are of mineral origin and result from the progressive abrasion of rocks. They are essentially made of quartz, silicon oxide, clay, and carbonates. They contain iron and are also covered with organic matter (bacteria and viable spores, grains of pollens) [8]. The experimental studies performed on rats revealed the toxic and inflammatory potential of desert dust for airways [9]. Epidemiologic studies have shown that this desert particulate pollution increased morbidity and mortality as well as aggravated the condition of patients suffering from chronic respiratory diseases [10], [11]. Few studies exist on the connections between Saharan dust and asthma. For example, in the Caribbean, only two studies have focused on the effects of Saharan dust on asthma in children and have provided contradictory results [12], [13].

This study concerns the city of Pointe-a-Pitre and its suburbs located on the archipelago of Guadeloupe, which, each year, is exposed to Saharan dust during several months.

We have studied, over one year, the effects of particulate pollutants (PM_10_, PM_2.5–10_) contained inside Saharan dust on the aggravation of asthma in children, by considering as a criterion, the number of visits of asthmatic children to the paediatric emergency department of the main medical facility of the archipelago. Our main assumption was based on a possible association between the intrusion of dust from the Sahara on the territory and the visits of asthmatic children to the emergency department.

## Materials and Methods

### A Ethics Statement

This study has been approved by the Institutional Review Board of the French Learned Society for Respiratory Medicine (Société de Pneumologie de Langue Française; CEPRO: 2013/018). Due to the fact that the data file has been anonymised, the name of the participants was not necessary for the analysis, and therefore we did not collect the participants’ names. The evaluation committee for observational research (CEPRO ) of the Institutional Review Board of the French Learned Society for Respiratory Medicine (Respiratory Society of French Language) estimated that this study was purely observational and consent written and informed consent of participants was not necessary because the research involves no intervention or contact with the patient.

### B/Study Area

The study area concerned the suburbs of the city of Pointe-a-Pitre, including all of the Grande Terre area, a region of the archipelago of Guadeloupe (16° North latitude and 61° West longitude), which is a French department located in the Caribbean. This area has a regular relief and is a large limestone plateau. It has a surface of 588 km^2^ with 197,603 inhabitants (Nation Institute of Statistics and Economic Surveys (INSEE); 2011) and a density estimated at 336 inhabitants/km^2^. The suburbs are crossed by roads but do not include any heavy industries.

### C/Population Under Study

The sanitary data were collected by the university hospital centre of Pointe-a-Pitre, the main medical structure of the department, which receives more than 65,000 visits in the emergency department each year, including 20,000 children. This centre is equipped with IT tools (IT extraction software) which are used for collecting medical data (visits to the emergency department). The daily visits due to the aggravation of asthma were codified according to the international classification of diseases (CIM 10^th^ edition) (J45–J46). The information provided by data extraction corresponded to the number of visits per day for asthma conditions to the paediatric emergency department, as well as administrative data concerning the age and gender of the children admitted in the health emergency structure. The study was carried out from January 1, 2011 to December 31, 2011 and concerned asthmatic children aged between 5 and 15 years old (included).

### D/Exposure Data

The days on which the dust intruded on the territory (index days) could be detected thanks to American meteorological satellite data available in real time on the following website: “Aerosol looper” [14]. The exposure data for the pollutants were provided by the regional agency approved by the public authorities for the quality of air, established at Pointe-a-Pitre. The agency has 4 measuring stations (3 fixed urban and peri-urban stations and 1 mobile stations), which regularly measure the following pollutants: PM_10_ (particles suspended in the air, with a median diameter lower than 10 micrometres, PM_2.5_ (particles suspended in the air, with a median diameter lower than 2.5 micrometres, sulphur dioxide (SO_2_), nitrogen dioxide (NO_2_), the nitric oxide (NO) and ozone (O_3_).

The P_2.5–10_ particles were calculated by subtracting the values of the PM_10_ and PM_2.5_ particles. For each pollutant, the daily average was determined by calculating the arithmetic average of the time values measured between 0.00 and 24.00. For ozone, the maximum value of the rolling averages over 8 hours was chosen.

The daily climatic parameters were provided by the regional meteorological station located at the airport of the city of Pointe-a-Pitre. The periods of maximum pollen emissions were determined according to the pollinic calendar of the department. The pollen calendar for the Pointe-à Pitre region (Guadeloupe) is a provisional calendar. The pollen characterization and count was carried out in 2004 by the Palynology lab of the Higher National School of Aerobiology (ENSA) in Montpellier, France. The responsible taxa corresponded to the Poaceae family (cereal) for 43% and to the Mimosaceae family for 16% of the cases. The pollination, taking into account the climate, is perennial in Guadeloupe. The responsible taxa represent the majority of emissions. The maximum emissions correspond to a period of around one week over a month. In the absence of a daily count of pollen on our territory, we only considered periods involving maximum emission of taxa.

The data on influenza epidemics were obtained by consulting the regional agency for monitoring influenza on the territory. The Regional Flu Monitoring Agency uses the number of flu consultations collected by the network of sentinel physicians practicing in the territory of Guadeloupe and from all the emergency cases at the region’s hospitals. An epidemic is declared when the epidemic threshold set by the agency on the percentage increase in the number of flu consultations is reached (2 to 3% per week).

### E/Statistical Analysis

Descriptive statistics were employed to describe all of the variables of this study for periods with and without the intrusion of Saharan dust.

The averages of the daily concentrations in pollutants, the daily average of climatic variables and the average number of visits for asthma per day to the emergency department were compared by means of the Student’s t-test or Mann-Whitney’s test for days with and without intrusions of Saharan dust.

Pearson’s correlations were calculated for the PM_10_, PM_2.5–10_ and PM_2.5_.

The chronological series, for particulate and gaseous pollutants, were produced graphically in order to observe their temporal distribution during the study period.

The frequency of visits due to asthmatic conditions per month during the study period was illustrated graphically.

The association between the daily concentrations in PM_10_ and PM_2.5–10_ and the daily visits due to asthmatic conditions in children were analysed by means of a time-stratified case-crossover study [15].

In this type of approach, each case has its own control: the exposure of a subject during the sanitary event (case period) is compared to the exposure of the same subject during one or several different moments (control period) where, a priori, the subject did not present the medical condition [16].

In agreement with the methods of this approach, the control periods were selected so as to correspond to the same day of the week and to the same month as the case day in order to minimise possible bias concerning trends and seasonality of the time series [16].

The effects of an exposure to the PM_10_ and PM_2.5–10_ were examined the same day (lag0) and up to 2 days before the exposure (lag2) but also by averaging the pollutant concentrations corresponding to two days before, one day before until the day of the event (lag0–2), (lag0–1).

We carried out a multivariate conditional logistic regression to estimate the odds ratio (ORs) by adjusting, on the climatic variables (temperature, humidity), the days of influenza epidemics (binary variable), and the days of maximum taxa emissions (binary variable) on bank holidays and during holidays (binary variable).

A binary variable was created for the days with and without Saharan dust. An interaction term between the average concentrations in PM_10_ and PM_2.5–10_ and the presence of Saharan dust was introduced into the modelling.

The analysis of sub-groups was based on the age categories (5 to 8 years old, 9 to 11 years old and 12 to 15 years old) and on the gender of the children (male or female).

The results were presented in the form of an excess risk percentage (IR %) with a confidence interval at 95% (CI 95%) for visits due to asthmatic conditions to the emergency department for an increase of 10 µg/m^3^ of PM_10_ and PM_2.5–10_ pollutants. These results were produced during two periods, with and without Saharan dust. The calculation of the IR % for an increase of 10 µg/m^3^ for PM_10_ and PM_ 2.5–10_ has been carried out using the following formula: (exp^ (ß*10)^ −1) ×100%, where ß is the model estimate. We also tested a bi-pollutant model with PM_2.5_ and PM_2.5–10_.

The statistical processing and analysis of data were carried out from an anonymised file using version 2.1.3.0 of R software. The significance threshold was set at 5% for all of the statistical tests.

## Results

### 1/Population Under Study

The study period included 337 days of observation, including 52 days (15% of the days of observation) involving the presence of Saharan dust (index days) and 285 days without Saharan dust.

During the study period, 836 visits to the emergency department took place in relation to asthmatic conditions in children aged between 5 and 15 years old. This figure included 58% (n = 489) of children aged between 5 and 8 years old, 27% (n = 222) of children aged between 9 and 11 years old and 15% (n = 125) of children aged between 12 and 15 years old. There were more boys (n = 514) than girls (n = 322). The sex ratio was of 1.6 in favour of the boys.

The ratio between boys and girls was comparable during the periods with and without Saharan dust.

**Figure 1:**
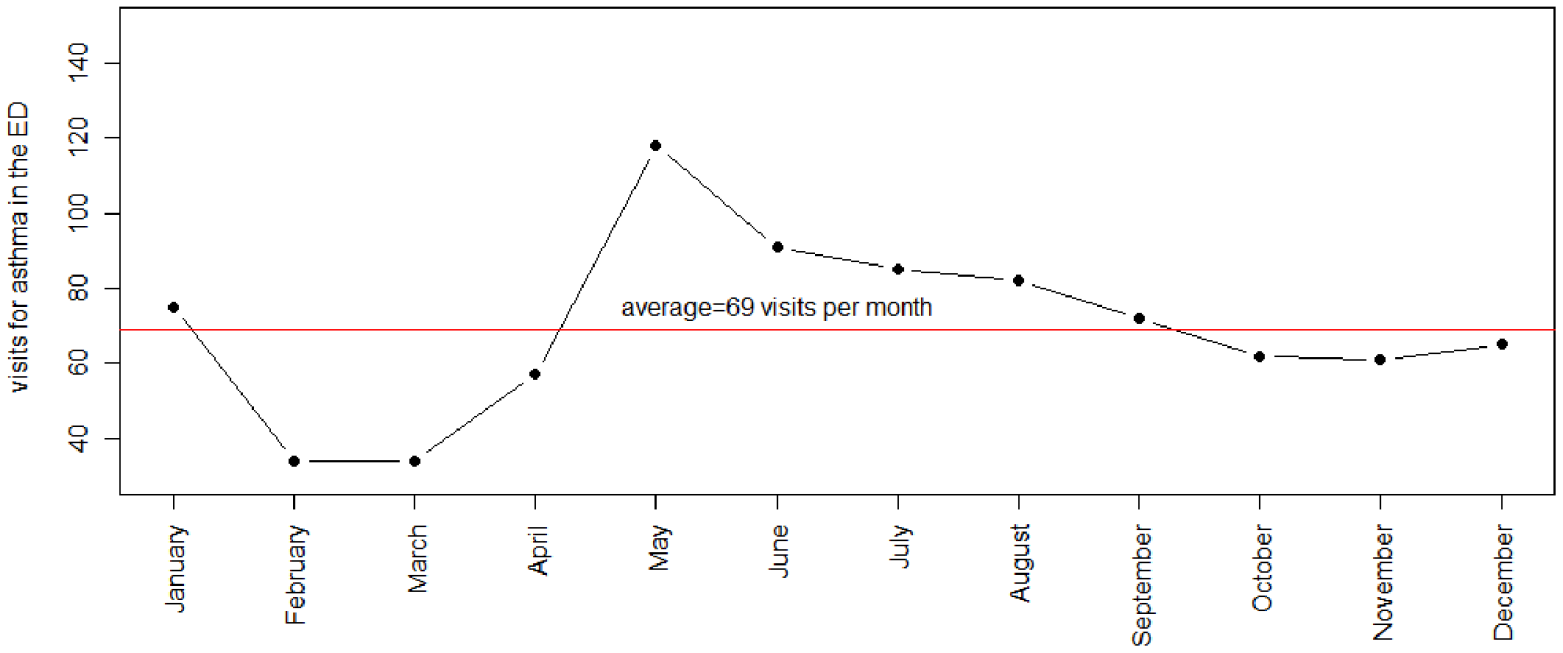
Frequency of visits for asthmatic conditions per month in the pediatric emergency department (ED) during the study period (n = 337 days).

Figure 1 shows the frequency of visits due to asthmatic conditions per month during the study period. The number of visits to the emergency department was higher from May to September.

Table 1 indicates the average and the number of visits per day to the emergency department due to asthmatic conditions for all children (5 to 15 years old) and for each age section being studied (5 to 8 years old, 9 to 11 years old, 12 to 15 years old) during periods with and without Saharan dust. The number of visits to the emergency department due to asthmatic conditions amounted to 220 visits for all of the children for the 52 days during which the presence of Saharan dust was detected and to 616 visits for the 285 days without Saharan dust.

**Table 1:** Daily visits for asthma in emergency department (ED) during Saharan dust-affected days and Saharan dust-free days in a study period.

Daily visits for asthma in ED during Saharan dust-affected days (52 days)						
	Mean		(SD)	Min	Median	Max
**For all children** **5–15 years** **n = 220** **Male = 132** **Female = 88**	4.2		(1.9)	0.0	3.0	10.0
**For children** **5–8 years** **n = 97** **Male = 58** **Female = 39**	1.8		(1.4)	0.0	1.0	7.0
**For children** **9–11 years** **n = 82** **Male = 49** **Female = 33**	1.5		(0.9)	0.0	0.0	5.0
**For children** <1p id="para73">**12–15 years** **n = 41** **Male = 25** **Female = 16**	0.7		(0.9)	0.0	0.0	4.0
**Daily visits for asthma in ED during Saharan dust-free days (285 days)**						
	**Mean**		**(SD)**	**Min**	**Median**	**Max**
**For all children** **5–15 years** **n = 616** **Male = 362** **Female = 254**		2.1	(1.8)	0.0	2.0	8.0
**For children** **5–8 years** **n = 392** **Male = 231** **Female = 161**		1.3	(1.5)	0.0	1.0	6.0
**For children** **9–11 years** **n = 140** **Male = 87** **Female = 53**		0.4	(0.6)	0.0	0.0	2.0
**For children** **12–15 years** **n = 84** **Male = 46** **Female = 38**		0.2	(0.5)	0.0	0.0	2.0

For all the children (5 to 15 years old), the average number of visits per day was higher during periods with Saharan dust compared to periods without Saharan dust (4.2±1.9 visits/day versus 2.1±1.8 visits/day; p = 0.02).

### 2/Data Concerning the Levels of Pollutants under Study and the Other Parameters of the Study: Climatic Variables, Periods of Influenza, Periods of Maximum Pollen Emissions

**Figure 2:**
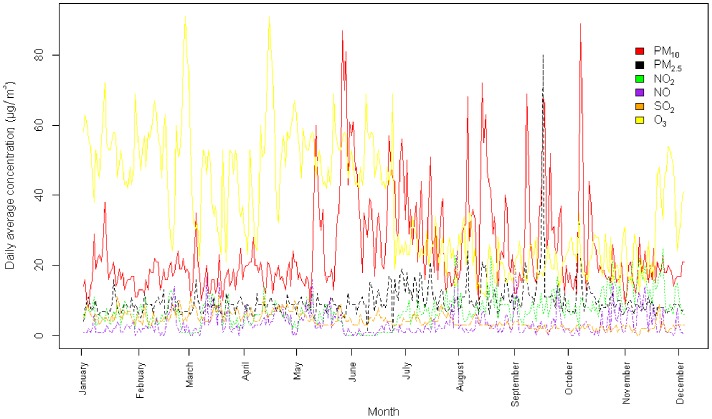
Temporal distribution of pollutants (PM_10_, PM_2.5_, NO_2_, NO, SO_2_, O_3_) during the study period (n = 337 days) in Guadeloupe. Abbreviations: PM_10_ particles with an aerodynamic diameter of 10 µm or less, PM_2.5_ particles with an aerodynamic diameter of 2.5 µm or less, NO_2_ nitrogen dioxide, NO nitrogen oxide, SO_2_ sulphur dioxide, O_3_ ozone.

Figure 2 illustrates the temporal distribution of the average daily concentrations in pollutants during the months of the study for the following pollutants: PM_10_, PM_2.5_, NO_2_, NO, SO_2_, O_3_.

Table 2 describes the average daily concentrations in pollutants and average measurements of climatic parameters for periods with Saharan dust (n = 52 days) and periods without Saharan dust (n = 285 days).

**Table 2:** Descriptive statistics of particulate matter, gaseous pollutants and meteorological variables during a period with Saharan dust-affected days and Saharan dust-free days.

Environmentalvariable	Mean	(SD)	Min.	p25	Median	p 75	Max
**Period with Saharan dust-affected days (n = 52 days)**							
**Particulate matter**							
**PM_10 (µg/m_^3^_)_**	49.7	(13.4)	36.0	38.0	47.7	57.0	89.0
**PM _2. 5__(µg/m_^3^_)_**	14.4	(10.5)	1.0	9.0	13.0	17.0	70.0
**PM _2.5–10 (µg/m_^3^_)_**	36.2	(14.1)	12.0	27.0	32.0	43.0	81.0
**Other pollutants**							
**NO_2 (µg/m_^3^_)_**	5.3	(1.6)	0.0	1.2	6.0	8.0	23.0
**SO_2 (µg/m_^3^_)_**	3.7	(1.4)	2.0	3.0	3.0	4.0	9.0
**O_3 (µg/m_^3^_)_**	33.4	(10.5)	11.5	19.6	26.3	48.0	72.0
**Weather**							
**Temperature** **(C°)**	28.9	(0.9)	26.9	28.0	29.0	30.0	31.0
**Relative humidity (%)**	76.8	(4.5)	69.0	73.0	76.8	79.0	92.0
**Period with Saharan dust-free days (n = 285 days)**							
**Particulate matter**							
**PM_10 (µg/m_^3^_)_**	19.2	(5.6)	8.0	16.0	18.0	21.5	34.0
**PM _2. 5__(µg/m_^3^_)_**	8.8	(2.4)	1.0	7.0	9.0	10.0	21.0
**PM _2.5–10 (µg/m_^3^_)_**	10.3	(5.3)	0.0	7.0	10.0	13.0	29.0
**Other pollutants**							
**NO_2__(µg/m_^3^_)_**	7.6	(4.5)	0.0	5.0	7.0	10.0	25.0
**SO_2(µg/m_^3^_)_**	4.3	(2.2)	2.0	3.0	4.0	6.0	12.0
**O_3 (µg/m_^3^_)_**	38.5	(10.2)	12.5	23.2	37.1	53.0	91.0
**Weather**							
**Temperature** **(C°)**	26.7	(1.6)	22.0	26.0	27.0	28.0	30.0
**Relative humidity (%)**	77.5	(5.5)	57.0	74.0	77.5	82.0	93.0

p25∶25th percentile.

p75∶75th percentile.

SD: standard deviation.

The average daily concentrations in particulate pollutants were higher during days with Saharan dust compared to days without Saharan dust: PM_10_ (49.7±13.4 µg/m^3^ versus 19.2±5.6 µg/m^3^; Student’s t tests, p = 0.001), PM_2.5–10_ (36.2±14.1 µg/m^3^vs.10.3±5.3 µg/m^3^; p = 0.001). PM_2.5_ (14.4±10.5 µg/m^3^ vs.8.8±2.4 µg/m^3^; p = 0.001).

The PM_2.5_/PM_10_ ratio was of 0.2 on average on days with Saharan dust and of 0.4 on days without Saharan dust.

For periods involving Saharan dust, the percentage of days with an average daily concentration in PM_10_ exceeding 50 µg/m^3^ was of 38% (n = 20 days).

The average daily concentrations in NO_2,_ SO_2_ and O_3_ pollutants were lower during days with Saharan dust compared to days without Saharan dust (p = 0.001, p = 0.001, p = 0.02, respectively).

PM_2.5_ and PM_2.5–10_ were moderately correlated (Pearson’ S correlation coefficient = 0.24) while PM_2.5_ and PM_10_ and PM_2.5–10_ and PM_10_ were highly correlated (r = 0.64 and r = 0.96, respectively).

The temperature was higher during the days with Saharan sand (p = 0.001), while the humidity was significantly comparable during the two study periods (p = 0.11).

The periods involving maximum pollen emissions represented 48% (n = 163 days) of all of days of observation (n = 337 days) and corresponded to 50% (n = 26 days) of the days with Saharan dust and 47% (n = 134 days) of the days without Saharan dust, without any significant difference in proportions between the two periods (p = 0.55). The relevant taxa were from the Poaceae family for 43% and from the Mimosaceae family for 16%.

Periods involving influenza represented 20% (n = 69 days) of days of observation. They concerned 7% (n = 4 days) of days with Saharan dust and 22% (n = 65 days) of days without Saharan dust with a significant difference in proportions between the two periods (p = 0.01).

### 3/Relation between Saharan Dust Episodes and the Number of Visits to the Paediatric Emergency Department Due to Asthmatic Conditions

**Figure 3:**
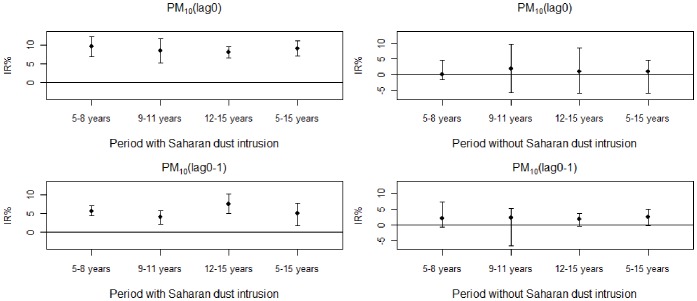
Percentage increase (IR %) of asthma-related visits to the ED for an increase of 10 µg/m^3^ of PM_10_ on the day of the visit (lag 0) or the previous 0 to 1 days (lag (0–1)) in each subgroup of children (aged 5 to 8 years old, 9 to 11 years old, 12 to 15 years old and 5 to 15 years old) during periods with and without Saharan dust intrusions. Error bars represent 95% confidence intervals.

**Figure 4:**
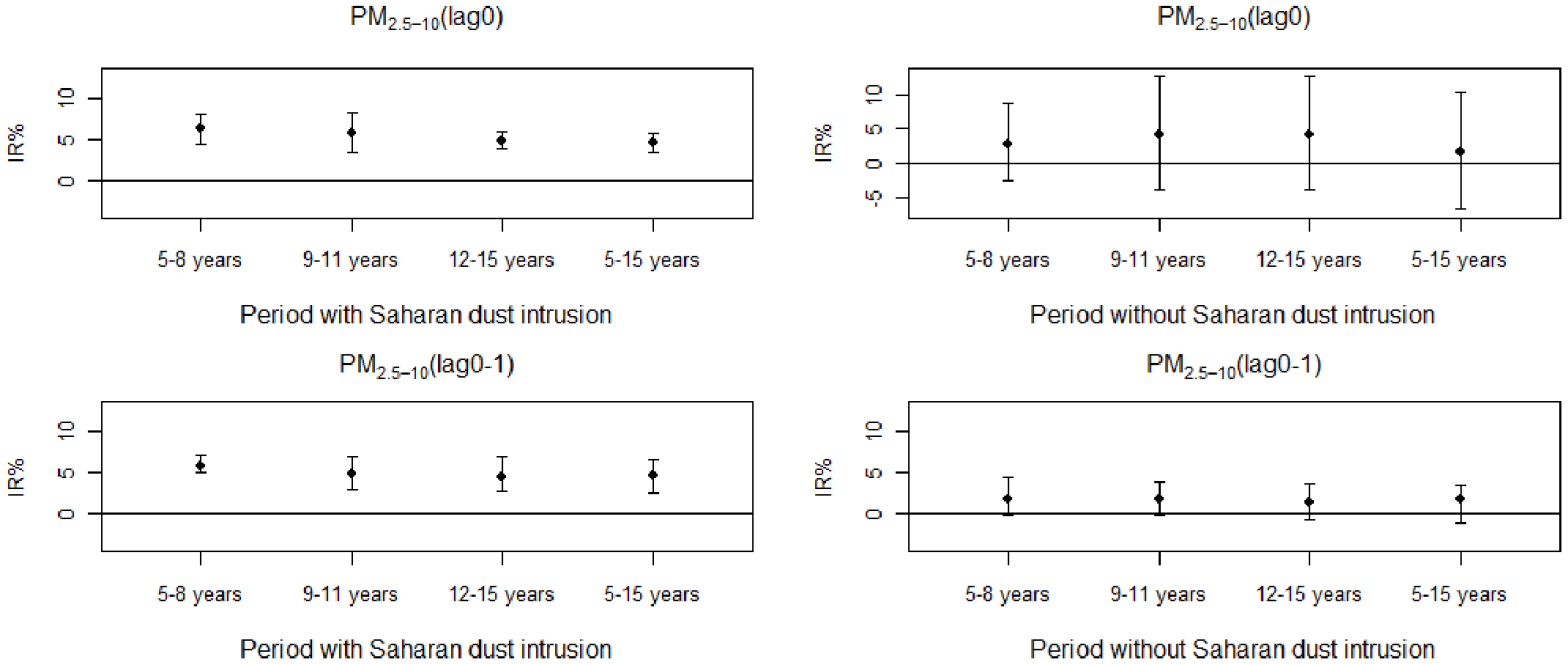
Percentage increase (IR%) of asthma-related visits to the ED for an increase of 10 µg/m^3^ of PM_2.5–10_ on the day of the visit (lag 0) or the previous 0 to 1 days (lag (0–1)) in each subgroup of children (aged 5 to 8 years old, 9 to 11 years old, 12 to 15 years old and 5 to 15 years old) during periods with and without Saharan dust intrusions. Error bars represent 95% confidence intervals.

Figures 3 and 4 graphically represent the IR% of daily visits with a CI 95% for asthmatic conditions in sub-groups of children aged between 5 and 8 years old, 9 and 11 years old, 12 and 15 years old and all of the children aged between 5 and 15 years old for an increase of 10 µg/m^3^ of pollutants PM_10_ and PM_2.5–10_ at lag 0 and at lag (0–1) during periods with and without intrusions of Saharan dust.

A statistically significant association is determined at lag0 and lag (0–1) for PM_10_ and PM_2.5–10_ and the visits due to asthmatic conditions during periods involving intrusions of Saharan dust, adjusted in relation to climatic parameters, periods of influenza and maximum pollen emissions, bank holidays and school holidays.

No statistically significant association was determined with pollutants PM_10_ and PM _2.5–10_ at lag0 and lag (0–1) for the visits of children suffering from asthma during the period without Saharan dust.

The IR% with a CI95% for an increase of 10 µg/m^3^ in pollutants PM_10_ and PM_2.5–10_ for periods with and without Saharan dust presented the following values (table S1 and S2).

For PM_10_ at lag 0:

For all children aged between 5 and 15 years old: the IR% was, during periods with and without Saharan dust, of 9.1% (CI95%, 7.1%–11.1%) versus 1.1% (CI95%, −5.9%–4.6%), respectively. The interaction between the index days and the association between the pollutant and the sanitary variable was significant (p value = 0.0012).

For the sub-groups of children during the periods with and without Saharan dust respectively: The IR% was, for children aged between 5 and 8 years old, of 9.5% (CI95%, 6.8%–12.2%) versus 0.1% (CI95%, −1.4%–4.6%), for children aged between 9 and 11 years old, of 8.4% (CI95%, 5.2%–11.7%) versus 1.9% (CI95%, −5.5%–9.7%), and for children aged between 12 and 15 years old of 8.0% (CI95%, 6.4%–9.6%) versus 1.1% (CI95%, −5.9%–4.6%).

For PM_10_ at lag (0–1):

For all children aged between 5 and 15 years old:

The IR % was, during periods with and without Saharan dust, of 5.1% (CI95%, 1.8%–7.7%) versus 2.4% (CI95%, −0.3%–5%), respectively.

For the sub-groups of children during the periods with and without Saharan dust respectively: The IR% for children aged between 5 and 8 years old was of 5.7% (CI95%, 4.4%–7.1%) versus 2.0% (CI95%, - −0.7%–7.2%), for children aged between 9 and 11 years old, of 4.0% (CI95%, 2.2%–5.8%) versus 2.2% (CI95%, −6.8%–5.3%), and for children aged between 12 and 15 years old, of 7.5% (CI95%, 5.0%–10.3%) versus 1.7% (CI95%, −0.4%–3.7%).

For PM_2.5–10_ at lag 0:

For all children aged between 5 and 15 years old: the IR% was, during the periods with and without Saharan dust, of 4.5% (CI95%, 3.3%–5.7%) versus 1.6% (CI95%, −6.5%–10.4%), respectively. The interaction between the index days and the association between the pollutant and the sanitary variable was significant (p value = 0.002).

For sub-groups of children during periods: with and without Saharan dust, the IR% was, for children aged between 5 and 8 years old, of 6.2% (CI95%, 4.4%–8.1%) versus 2.9% (CI95%, −2.5%–8.7%), for children aged between 9 and 11 years old, of 5.7% (CI95%, 3.3%–8.2%) versus 4.3% (CI95%, −3.7%–12.8%), for children aged between 12 and 15 years old, of 4.8% (CI95%, 3.8%–5.9%) versus 4.3% (CI95%, −3.7%–12.8%), respectively.

For PM_2.5–10_ at lag (0–1):

For all children aged between 5 and 15 years old: the IR% was, during periods with and without Saharan dust, of 4.7% (CI95%, 2.5% –6.5%) versus 1.8% (CI95%, −1.1%–3.4%), respectively.

For sub-groups of children during periods with and without Saharan dust: the IR% was, for children aged between 5 and 8 years old, of 5.9% (CI95%, 5.0%–7.2%) versus 1.7% (CI95%, – 0.1%–4.4%), for children aged between 9 and 11 years old, of 4.9% (CI95%, 3.0%–6.9%) versus 1.8% (CI95%, −0.2%–3.8%), for children aged between 12 and 15 years old, of 4.4% (CI95%, 2.8%–7.0%) versus 1.4% (CI95%, −0.7%–3.6%), respectively.

**Figure 5:**
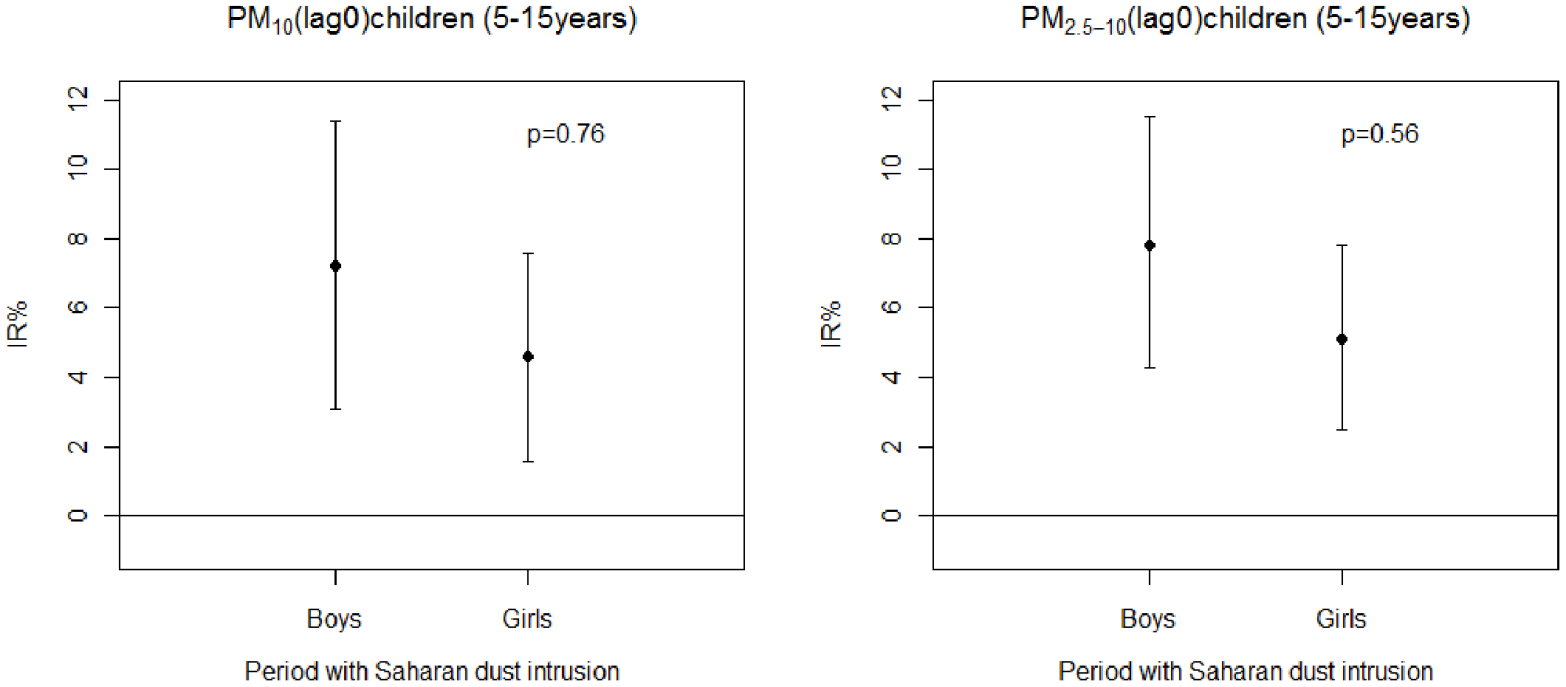
Percentage increase (IR %) of asthma-related visits to the ED for an increase of 10 µg/m^3^ of PM_10_ and PM_2.5–10_ on the day of the visit (lag 0) in each subgroup of children (boys and girls) during a period with Saharan dust intrusions. Error bars represent 95% confidence intervals.

Figure 5 graphically represents the IR% of daily visits with a CI 95% due to asthmatic conditions per gender and for all children (5 to 15 years old) and for an increase of 10 µg/m^3^ in pollutants PM_10_ and PM_2.5–10_ at lag 0 for periods with Saharan dust:

For PM_10_: the IR% was of 7.2% (CI95%, 3.1%–11.4%) for boys and of 4.6% (CI95%, 1.6%–7.6%) for girls.

For PM_2.5–10_: the IR% was of 7.8% (CI95%, 4.2%–11.5%) for boys and of 5.1% (CI95%, 2.5%–7.8%) for girls.

There was no statistical difference in the IR% for periods with Saharan dust among different age group of children (Table S3, S4) and between boys and girls (Figure 5) for PM_10_ and PM_2.5–10_ at lag0 and at lag (0–1).

The other delays (lag1, lag2, lag0, 2) tested for pollutants PM_10_ and PM_2.5–10_ during the study period for all children and for the sub-groups were not contributory. Results are reported in the table S5.

No significant effect was revealed for the PM_2.5_ during periods with and without Saharan dust at different delays. (Table S5).

A bi-pollutant model was tested with pollutants PM_2.5_ and PM_2.5–10._ Excess risks for PM_2.5–10_ were not significantly modified for all children at lag 0 and at lag (0–1).

## Discussion

This study highlighted a statistically significant association between the PM_10_ and PM_2.5–10_ pollutants contained in the Saharan dust and the visits made to the emergency department due to asthmatic conditions in children aged between 5 and 15 years old during the period involving Saharan dust intrusions.

This association resulted in an excess risk, on the actual day of exposure to the pollutants, of 9.1% (CI95%, 7.1%–11.1%) for the PM_10_ and of 4.5% (CI95%, 2.5%–6.5%) for the PM_2.5–10_ on days involving Saharan dust intrusions with a significant interaction with the pollutants.

The level of average daily concentrations in PM_10_ in this study was comparable to that of the studies performed on pollution due to Saharan dust in Madrid (Spain) [11] or in Rome (Italy) [17]. The level of average concentrations in PM_2.5–10_ on days with Saharan dust was relatively higher in our study compared to the study carried out in Madrid (average = 36.2 µg/m^3^ versus 24.2 µg/m^3^) [11], because the levels of concentration in PM_2.5_ had lower values in our study (14.4 µg/m^3^ versus 24.4 µg/m3). The former studies carried out in the Caribbean did not provide measurements of the concentrations in pollutants contained in the Saharan dust on days of pollution [12], [13]. The study performed by Gyan et al. only measured optical visibility whereas the study carried out by Prospero et al. was based on the measurement of the total rate of dust during Saharan dust intrusions.

As regards the level of gas pollution, for example for pollutant NO_2_ in our study, the level of average daily concentrations was 10 times lower than the level recorded in Madrid (5.3 µg/m^3^ versus 60.5 µg/m^3^) [11]. Throughout the study period, gas pollution (NO_2_, SO_2_) and chemical pollution (O_3_) remained at low levels of daily concentrations and the thresholds set by the WHO were never exceeded. This can be explained by the absence of heavy industries on the archipelago and by the all-year-round presence of trade winds, which dispersed the gaseous and chemical pollutants.

The chemical composition of the Saharan dust includes mineral elements: primarily quartz (60%), oxides (SiO_2_, FeO_2_) and carbonates (CaCO_3_), but also iron, titanium and vanadium [7]. In certain cases, the dust intrusion episodes can involve peaks in sulphate resulting from the chemical reaction between the carbonates contained in the dust and the gaseous pollutants present locally (NO_2_, SO_2_) [7]. Toxicological studies reported that the quartz, crystalline silica, aluminium and oxides contained in desert dust could cause an inflammation of the bronchi and lungs in rats due to a hyperproduction of cytokines [9], [18].

Studies have shown that the Saharan dust introduced, into the atmosphere, viable micro-organisms and other microbiological matter (pollen, lipopolysaccharide (LPS), viable mushrooms, mould, viruses, and bacteria) [8]. Several authors have reported an excess risk of mortality due to respiratory causes during Saharan dust intrusions [11], [17]. It has been known for a long time that pollutants, and especially particulate pollutants, aggravate asthmatic conditions in children [19]. It was reported that asthmatic children were more sensitive to air pollutants than non-asthmatic children [20].

In the Caribbean, children’s asthma is significantly predominant and the reasons are still unknown [21]. In Guadeloupe, the ISAAC study reported a prevalence of asthma in children of 14.1%, which was higher for boys than for girls (15% versus 13.1%) [3].

In our study, the population being analysed was important (n = 836 children) with a prevalence of boys (60% overall), which complied with the epidemiologic studies on asthma in children performed in our area. These results are also in agreement with the epidemiologic studies on asthma in children, which indicated a major incidence of asthma among boys [22].

The determination of an association between the visits to the emergency department due to asthmatic conditions and Saharan dust intrusions was carried out in this study based on a time-stratified case-crossover methodology. This approach offers several advantages:

It can be used for controlling the individual confounding factors as each case is its own control [16], which is interesting in relation to asthma where individual susceptibilities are to be considered and controlled. In addition, it also makes it possible to take into account individual characteristics such as age or gender to explore the effects of pollutants on the different sub-groups [23]. Moreover, it can be used to control trends and seasonality [24].

This study has highlighted an increase in risks with pollutants PM_10_ and PM_2.5–10_ during periods involving Saharan dust intrusions. The effects were more marked with the PM_10_ than with the PM _2.5–10_ with a significant interaction between these particles and the index days. These effects were maximum and significant on the actual day of pollution. However, we did not reveal any effect with the PM_2.5_ on the visits for asthmatic conditions regardless of the period being analysed; this could be explained by the low level of concentrations in PM_2.5_ in this study, adjusted to a low relative anthropic pollution observed on the archipelago [25].

Samoli et al. objectified, in Athens, an increase in admissions for asthmatic conditions of 2.54% with the PM_10_ during periods involving Saharan dust intrusions although the interaction between the presence of desert dust and the concentrations in PM_10_ was not significant. This author also observed a maximum effect of the PM_10_ on the actual day of exposure [26].

A study performed in Toyama, in Japan, observed an association between desert dust from Mongolia and China and the hospitalisation of children due to aggravated asthmatic conditions, by using a quantitative measurement of mineral dust in air [27]. The risk of hospitalisation for asthmatic conditions was high for boys and the youngest children, but in this study, only 6 days involved desert dust intrusions [27]. In our study, the effects of the PM_10_ were marked, on days involving Saharan dust intrusions, on young children (5 to 8 years old) while the PM_2.5–10_ had a pronounced effect on teenagers (12 to 15 years old). Both pollutants had an effect on boys and girls. Several explanations can be proposed: young children have a greater tendency to breathe through their mouth than their nose and we know that the breathing process (nasal, oral) is an important factor in the deposit and concentration of pollutants at the level of the bronchial tree [28]. In addition, their respiratory frequency is high and they have a greater pulmonary surface per weight unit than an adult [29]. Moreover, they spend more time outside and still have an immature immune system. In addition, boys, especially the youngest, are hyperactive [30]. Our results are consistent with other studies, which reported that the effects of the pollutants were significant among boys and young children [22].

The difference between the effects of the PM_10_ and PM_2.5–10_ contained in dust on different age sub-groups is difficult to explain. A possible assumption concerns the location and quantity of dust deposit through the respiratory system. Venkataraman et al. showed that, on the basis of the mass of the particles, their deposit in the lungs and in the bronchi, was much higher with the PM_10_ than with the PM_2.5–10_ [31]. The inflammatory potential of particles PM_2.5–10_ is as experimentally high as for the PM_10_ [32]. Lin et al. observed an increase in hospitalisations for asthmatic conditions in the presence of the PM_2.5–10_ [33].

In the Caribbean, two studies with the contradictory results were undertaken with Saharan dust:

The first study was carried out on the island of Trinidad, it used optical visibility to measure the density of dust and revealed, by using a Poisson regression model, a significant association between the decrease in visibility and the admissions of children due to asthmatic conditions adjusted to climatic variables [12]. The second study carried out in Barbados did not show any association between Saharan dust and the number of admissions for asthmatic conditions in the main hospital of the island [13].

The disparity of the observations in the Antilles seems to suggest a strong dependency on the surrounding environment. The polluting industries, automobile traffic, and natural presence of pollen or aerosols in suspension in the atmosphere create local particularities specific to each island of the Caribbean. The intrusion of Saharan dust can therefore have different impacts, which are more or less significant, on the health of the population being exposed [34].

In the United States, a study determined an association between the PM_10_ from desert dust and hospital admissions due to respiratory causes in Washington [35]. Another study performed in Anchorage (USA) highlighted an increase in hospital visits due to asthma, bronchitis and high respiratory infections with PM_2.5–10_ particles from desert dust [36].

In Asia, two studies carried out in Korea [37], [38], showed the significant effects of desert dust on asthmatic children, while another study, carried out in Taiwan [39], did not find any connection. In Australia, an impact on severe asthma conditions was highlighted during dust periods [40].

There still are a limited number of studies, which have been carried out on desert dust. A majority of studies support the possibility that pollutants contained in desert dust have an effect on asthmatic children. However, these studies are difficult to compare because the exposure measurements are not sufficiently described and the confounding factors are not controlled sufficiently [41].

The strong point of our study is that it provides a complete description of the pollutants during periods with and without Saharan dust intrusions and it analyzes the effects of Saharan dust among groups of children of different age and gender in addition to pollutants. As far as we know, this study showed, for the first time, that particles PM_2.5–10_ contained in Saharan dust had an impact on the visits of children to the emergency department due to aggravated asthmatic conditions during the study period.

However, our study presents a certain number of limitations:

Firstly, this type of study is based on the assumption that all of the population under study was exposed to the same amount of pollutant, which cannot be verified. Moreover, it did not take into account the concentrations of pollutants actually inhaled by each child.

Furthermore, this study was carried out on a single site, which could lead to a selection bias.

In addition, the visits to the emergency department were counted by data-processing extraction according to the international classification of diseases (CIM 10th edition) J45–J46 and we cannot affirm the absence of errors in the codes.

Concerning pollen emissions, we used the pollinic calendar of the region, which is definitely less accurate than pollinic counting, which has not been implemented on the archipelago.

A bi-pollutant model was tested with the PM_2.5_ and PM_2.5–10_ but not between the PM_10_ and PM_2.5_ or PM_2.5–10_ due to the strong correlation existing between these pollutants. In addition, due to the fact that the gaseous and chemical pollutants did not show any connection with the sanitary variable, they were not tested by a bi-pollutant model.

Lastly, we did not obtain any data on the chemical or biological nature of the particles contained in the dust to confirm their mineral or anthropic origin.

## Conclusion

This study showed that on days involving Saharan dust intrusions, during the study period, the PM_10_ and PM_2.5–10_ particulate pollutants contained in the dust were responsible for an excess risk for visits to the emergency department due to aggravated asthmatic conditions in children aged between 5 and 15 years old. These results deserve to be confirmed by other studies on this topic and could have interesting repercussions, especially concerning the implementation of preventive or therapeutic strategies aiming to improve the treatment of asthmatic children during days involving Saharan dust intrusions.

## Supporting Information

Table S1 **The excess risk percentages (IR %) with 95% confidence intervals (CI) of visits to the pediatric emergency department due to asthmatic conditions (stratified by age of the children) for an increase of 10 µg/m^3^ of pollutants (PM_10_, PM_2.5–10_) on the day of visit (lag 0) during periods with and without Saharan dust.**
(DOCX)

Table S2 **The excess risk percentages (IR %) with 95% confidence intervals (CI) of visits to the pediatric emergency department due to asthmatic conditions (stratified by age of the children) for an increase of 10 µg/m^3^ of pollutants (PM_10_, PM_2.5–10_) on the previous 0 to 1 days of visit (lag 0–1) during periods with and without Saharan dust.**
(DOCX)

Table S3 **Comparison of IR% (excess risk percentages) between groups of children 5–8 years and 9–11 years during periods of Saharan dust for PM_10_ and PM_2.5–10_ at lag 0 and lag (0–1).**
(DOCX)

Table S4 **Comparison of IR% (excess risk percentages) between groups of children 5–8 years and 12–15 years during periods of Saharan dust for PM_10_ and PM_2.5–10_ at lag 0 and lag (0–1).**
(DOCX)

Table S5 **The excess risk percentages (IR %) with 95% confidence intervals (CI) of visits to the pediatric emergency department due to asthmatic conditions (for all the children (5–15 years) for an increase of 10 µg/m^3^ of pollutants (PM_10_ and PM_2.5–10_ (lag1, lag2, lag (0, 2), PM_2.5_ (lag0, lag1, lag (0, 1) lag2, lag0, 2)) during periods with and without Saharan dust.**
(DOCX)
