# Supplementary material for: Short-Term Effects of the Particulate Pollutants Contained in Saharan Dust on the Visits of Children to the Emergency Department due to Asthmatic Conditions in Guadeloupe (French Archipelago of the Caribbean)
Source: PLoS One. 2014 Mar 6;9(3):e91136. doi: 10.1371/journal.pone.0091136 (PMC3946322; doi:10.1371/journal.pone.0091136)
Supplement: Table S2 — The excess risk percentages (IR %) with 95% confidence intervals (CI) of visits to the pediatric emergency department due to asthmatic conditions (stratified by age of the children) for an increase of 10 µg/m3 of pollutants (PM10, PM2.5–10) on the previous 0 to 1 days of visit (lag 0–1) during periods with and without Saharan dust. (DOCX) [file pone.0091136.s002.docx]

| **Period** | **Period with Saharan dust (52 days)** | | **Period without Saharan dust (285 days)** | | | |
| --- | --- | --- | --- | --- | --- | --- |
| **Particulate matter** | **PM_10_ (lag0, 1)**  IR %*  (CI95%) p-Value | **PM_2.5-10_ (lag0, 1)**  IR% *  (CI95%) p-Value | **PM_10_ (lag0, 1)**  IR%*  (CI95%) p-Value | | **PM_2.5-10_ (lag0, 1)**  IR%*  (CI95%) p-Value | |
| **Age of children** |  | | | | | |
| **5-15 years** | 5.1  [1.8-7.7] 0.001 | 4.7  [2.5-6.5] 0.001 | | 2.4  [-0.3-5.0] 0.23 | | 1.8  [-1.1-3.4] 0.55 |
| **5-8 years** | 5.7  [4.4-7.1] 0.001 | 5.9  [5.0-7.2] 0.001 | | 2.0  [-0.7-7.2 ] 0.24 | | 1.7  [-0.1-4.4] 0.75 |
| **9-11 years** | 4.0  [2.2-5.8] 0.001 | 4.9  [3.0-6.9] 0.001 | | 2.2  [-6.8-5.3] 0.35 | | 1.8  [-0.2-3.8] 0.28 |
| **12-15 years** | 7.5  [5.0-10.3] 0.001 | 4.4  [2.8-7.0] 0.001 | | 1.7  [-0.4-3.7] 0.44 | | 1.4  [-0.7-3.6] 0.82 |

Table S2.

*Adjusted for temperature, relative humidity, influenza epidemics, periods of maximum issuance of pollen and holidays.
