# Supplementary material for: Short-Term Effects of the Particulate Pollutants Contained in Saharan Dust on the Visits of Children to the Emergency Department due to Asthmatic Conditions in Guadeloupe (French Archipelago of the Caribbean)
Source: PLoS One. 2014 Mar 6;9(3):e91136. doi: 10.1371/journal.pone.0091136 (PMC3946322; doi:10.1371/journal.pone.0091136)
Supplement: Table S3 — Comparison of IR% (excess risk percentages) between groups of children 5–8 years and 9–11 years during periods of Saharan dust for PM10 and PM2.5–10 at lag 0 and lag (0–1). (DOCX) [file pone.0091136.s003.docx]

Table S3.

| **Particulate matter** | **Age of children** | | **p-Value** |
| --- | --- | --- | --- |
|  | **5-8 years**  IR% CI95% | **9-11 years**  IR% CI95% |  |
| **PM_10_ lag 0** | 9.5 (6.8-12.2) | 8.4 (5.2-11.7) | 0.80 |
| **PM_10_ lag (0-1)** | 5.7 (4.4-7.1) | 4.0 (2.2-5.8) | 0.74 |
| **PM_2.5-10_ lag 0** | 6.2 (4.4-8.1) | 5.7 (3.3-8.2) | 0.99 |
| **PM_2.5-10_ lag (0-1)** | 5.9 (5.0-7.2) | 4.9 (3.0-6.9) | 0.98 |
