# Supplementary material for: Short-Term Effects of the Particulate Pollutants Contained in Saharan Dust on the Visits of Children to the Emergency Department due to Asthmatic Conditions in Guadeloupe (French Archipelago of the Caribbean)
Source: PLoS One. 2014 Mar 6;9(3):e91136. doi: 10.1371/journal.pone.0091136 (PMC3946322; doi:10.1371/journal.pone.0091136)
Supplement: Table S5 — The excess risk percentages (IR %) with 95% confidence intervals (CI) of visits to the pediatric emergency department due to asthmatic conditions (for all the children (5–15 years) for an increase of 10 µg/m3 of pollutants (PM10 and PM2.5–10 (lag1, lag2, lag (0, 2), PM2.5 (lag0, lag1, lag (0, 1) lag2, lag0, 2)) during periods with and without Saharan dust. (DOCX) [file pone.0091136.s005.docx]

**Table S5.**

|  | **Period with**  **Saharan dust** | **Period without**  **Saharan dust** |
| --- | --- | --- |
| **Particulate matter** | IR* (%)  (CI95%) p-Value | IR* (%)  (CI95%) p-Value |
| **PM_10_ (lag1)** | 4.1  [-0.1-8.7] 0.36 | 1.2  [-4.6-4.7] 0.57 |
| **PM_10_ (lag2)** | 1.4  [-1.2-8.5] 0.48 | 1.1  [-5.5-9.7] 0.57 |
| **PM_10_ (lag0,2)** | 1.2  [-6.4-9.2] 0.75 | 1.2  [-4.1-8.5] 0.76 |
| **PM_2.5-10_ (lag1)** | 3.3  [-0.2-9.7] 0.91 | 0.9  [ -2.2-4.7] 0.82 |
| **PM_2.5-10_ (lag2)** | 1.1  [-4.1-8.7] 0.85 | 1.0  [-5.5-9.9] 0.75 |
| **PM_2.5-10_ (lag0,2)** | 1.1  [-3.1-7.7] 0.82 | 1.3  [-5.2-8.6] 0.83 |
| **PM_2.5_ (lag0)** | 1.4  [-0.6-5.2] 0.55 | 1.1  [-4.5-7.7] 0.67 |
| **PM_2.5_ (lag1)** | 2.2  [-3.6-8.2] 0.45 | 1.9  [-2.5-8.7] 0.91 |
| **PM_2.5_ (lag0,1)** | 1.4  [-2.7-5.0] 0.54 | 1.3  [-4.1-6.5] 0.86 |
| **PM_2.5_ (lag2)** | 1.2  [-4.6-8.7] 0.76 | 1.0  [-4.2-8.6] 0.66 |
| **PM_2.5_ (lag0,2)** | 1.1  [-2.1-7.5] 0.83 | 1.1  [-3.1-8.5] 0.76 |
